# Novel NSAID-Se derivative YSN-1-167 against Enterovirus 71 infection by inhibiting 3D^pol^ activity

**DOI:** 10.1128/aac.01864-25

**Published:** 2026-05-29

**Authors:** Yangxin Qi, Weiling Li, Qian Peng, Yingying Shi, Nan Tan, Yanrong Yang, Yinying Zhu, Song Hu, Bo Zhang, Pin Wan, Xiji Shu, Yuchen Liu, Liming Liu, Xianran He, Binlian Sun

**Affiliations:** 1 Hubei Key Laboratory of Cognitive and Affective Disorders, School of Medicine, Institute of Biomedical Sciences, Jianghan University 470004, Wuhan, China; 2 Hubei Provincial Demonstration Center for Experimental Medicine Education, School of Medicine, Jianghan University 470004, Wuhan, China; 3 Department of Immunology, School of Medicine, Jianghan University 470004, Wuhan, China; 4 Key Laboratory of Virology and Biosafety, Center for Emerging Infectious Diseases, Wuhan Institute of Virology, Chinese Academy of Sciences 74614 , Wuhan, China; 5 Department of Hepatology, Hubei Third People’s Hospital, Wuhan, China; Chinese Academy of Medical Sciences & Peking Union Medical College, Beijing, China

**Keywords:** NSAID-selenocyanate derivatives, YSN-1-167, HFMD, EV71, 3D polymerase

## Abstract

Hand, foot, and mouth disease (HFMD) is primarily caused by Enterovirus 71 (EV71) and Coxsackievirus A16 (CA16), while excessive inflammatory responses induced by viral infection are the main cause of severe conditions. Hence, it is important to develop agents with both antiviral and anti-inflammatory activities for clinical treatment. In this study, we synthesized 20 compounds based on the hybridization of nonsteroidal anti-inflammatory drugs (NSAIDs) and organoselenium and found that YSN-1-167 exhibited significant anti-EV71 function using an EV71-GFP infection system. Mechanistic studies showed that this compound suppressed virus replication but not the binding and entry steps, and further target screening found that YSN-1-167 might suppress 3D polymerase function but not viral 2A and 3C proteases. The conservation of 3D polymerase between EV71 and CA16 prompted us to discover that YSN-1-167 can also significantly inhibit CA16 infection. As expected, this compound can reduce the levels of pro-inflammatory mediators, including IL-1β and COX-2, induced by EV71 infection. Furthermore, YSN-1-167 exhibited favorable safety and effective anti-EV71 and anti-inflammatory properties in neonatal mice. In conclusion, these results suggested that YSN-1-167 can be developed into a potential therapeutic strategy for HFMD induced by EV71 and CA16 via inhibiting virus replication and inflammatory response.

## INTRODUCTION

Hand, foot, and mouth disease (HFMD) is a contagious viral illness that typically causes skin rashes, herpes, and fever in infants and children under 5 years old. In addition to these common symptoms, some patients may develop central nervous system symptoms, such as aseptic meningitis, brainstem encephalitis, neurogenic pulmonary edema, and heart failure (1). Enterovirus 71 (EV71) and Coxsackievirus A16 (CA16) are generally considered to be the most common causative pathogens of HFMD, with EV71 being the most frequently identified serotype among severe and fatal cases (2). While the EV71 vaccine available in China can effectively reduce the incidence of EV71-associated HFMD, there are still cases of severe HFMD every year, and it does not induce cross-immunity to other enteroviruses (3, 4). Since effective vaccines are unavailable for most of these viruses, there is an urgent need for antiviral drug discovery. Currently, the prevention of HFMD epidemics relies primarily on public health awareness, and conventional therapies such as ribavirin and glucocorticoids are used in clinical practice (5); still, we have no specific antiviral agents available for EV71- and CA16-associated diseases.

EV71 belongs to the Human Enterovirus A species of the *Picornaviridae* family. The virus consists of a single-stranded, positive-sense RNA of approximately 7,500 nucleotides. The life cycle of EV71 involves attachment, entry, replication, and release. After viral entry, the internal ribosome entry site (IRES) within the 5′ untranslated region (5′-UTR) drives the translation of the viral polyprotein, which is encoded as the VP4-VP2-VP3-VP1-2A-2B-2C-3A-3B-3C-3D and is subsequently cleaved by viral 2A and 3C proteases into various structural and non-structural proteins. The 3D protein, also known as RNA-dependent RNA polymerase (RdRp), is the viral enzyme responsible for viral genomic RNA synthesis (6). These viral enzymes involved in viral replication are considered to be potential molecular targets for antiviral drug development (7).

Selenium is an essential trace element that plays a crucial role in human health. Previous reports have indicated that adequate selenium levels help sustain the activity of natural killer cells and the proliferation of T cells, which are important for antiviral immunity (8). Research has shown that selenium deficiency can increase the virulence of a human enterovirus such as Coxsackievirus B3 (9). In previous studies, selenium nanoparticles were shown to significantly enhance the anti-EV71 activity of oseltamivir in cellular models (10). Furthermore, the organoselenium compound ebselen was found to inhibit SARS-CoV-2 replication by forming an irreversible covalent bond between its selenium moiety and Cys145 in the catalytic dyad of the viral main protease (11). Although the antiviral mechanisms of organoselenium compounds vary across viruses, these findings suggest that they possess broad-spectrum antiviral potential.

EV71 infection activates the innate and adaptive immune systems, triggering the release of a large number of cytokines. This response helps the body to suppress virus replication but can also induce inflammatory tissue damage. The expression levels of TNF-α, IFN-γ, IL-6, and IL-1β in the serum of the severe symptoms group are significantly higher than those in the mild group and the control group (12). Elevated IL-1β levels have been observed in the serum of EV71-infected patients with encephalitis, as well as in mouse models (13). EV71 infection can enhance cyclooxygenases-2 (COX-2) and IL-1β expression via the AP-1 and NF-κB pathways in rat brain astrocytes (14). Nonsteroidal anti-inflammatory drugs (NSAIDs) are the most widely used medicines for suppressing the inflammatory response by inhibiting the activity of COX, thereby blocking the synthesis of prostaglandins (15). NSAIDs have been used to alleviate the inflammatory symptoms induced by viral infections (e.g., fever, cough, body aches, and pains) due to their analgesic, anti-inflammatory, and antipyretic activities (16).

Given the anti-inflammatory effects of NSAIDs and the antiviral potential of organoselenium compounds, we screened a library of NSAID-selenocyanate derivatives and identified YSN-1-167, which exhibited significant anti-EV71 and anti-CA16 activity, as well as anti-inflammatory activity. Mechanistic studies revealed that YSN-1-167 inhibits viral replication by suppressing 3D polymerase (3D^pol^) activity of EV71, potentially through interacting with the 3D^pol^ of EV71. These findings demonstrated YSN-1-167 as a promising lead compound for HFMD drug development.

## MATERIALS AND METHODS

### Cells, viruses, and plasmids

We utilized the following cell lines routinely maintained in our laboratory: human embryonal rhabdomyosarcoma (RD), human embryonic kidney (293T), human monocytic (THP-1), and African green monkey kidney epithelial (Vero) cells. RD, 293T, and Vero cells were cultured in DMEM supplemented with 10% fetal bovine serum (FBS), whereas THP-1 cells were maintained in RPMI 1640 medium with 10% FBS. All cell lines were incubated at 37°C with 5% CO_2_. Prior to experiments, THP-1 cells were differentiated into adherent macrophages by a 12-h treatment with 100 ng/mL phorbol 12-myristate 13-acetate (PMA, Sigma, P1585).

The EV71 expressing green fluorescent protein (EV71-GFP) was prepared from the infectious cDNA clone of EV71 (17). The wild-type EV71 strain (EV71-LYG03, LYG03/JS/CHN/2019, GenBank accession number: PQ015377) and a CA16 strain (CA16 #3, GenBank accession number: PX095330) used in this study were maintained in our lab. The influenza PR8 strain was a gift from Professor Jianjun Chen of Wuhan Institute of Virology, and the dengue virus was kindly provided by Associate Professor Jialu Qiao of Hubei University.

The genes encoding EV71 2A and 3D were cloned into the pcDNA3.1(+)−3× Flag vector to construct the expression plasmids for their expression in mammalian cells. The 2A protease activity-inactivated mutant C110S was constructed with a site-directed mutation by PCR using the plasmid pcDNA3.1(+)−3× Flag-2A as the template. Additionally, the genes for EV71 2A and 3C were cloned into the prokaryotic expression vector pET30a for subsequent protein purification. The luciferase reporter plasmids pFLuc and pGLuc were purchased from Beyotime Biotechnology (D2206 for pFLuc and D2098 for pGLuc), and the 5′-UTR of the EV71 genome was inserted into the upstream of the firefly luciferase (FLuc) gene in the pFLuc vector to generate a reporter construct. To create a 3D-dependent GLuc reporter plasmid for the specific detection of viral polymerase activity, the 3′-UTR and 5′-UTR of the EV71 genome were inserted into the flanking regions of the Gaussia luciferase (GLuc) gene in the pGLuc vector. All constructs generated in this study were verified by DNA sequencing performed by Sangon Biotech.

### Inhibitors and antibodies

The following compounds were used as positive controls: ribavirin (HY-B0434, 80 μM, MCE), a known nucleotide analog inhibitor of viral replication; sulindac (HY-B0008, 20 μM, MCE), an NSAID with broad-spectrum anti-inflammatory activity; aurintricarboxylic acid (ATA, A914659, 200 μM, Macklin), a known inhibitor of EV71 3D^pol^ activity (7); and sorafenib (HY-10201, 3 μM, MCE), which inhibits EV71 replication by targeting IRES-mediated translation (18).

The following antibodies were used as primary antibodies diluted 1:1,000: rabbit anti-β-actin (Proteintech, 81115-1-RR), rabbit anti-COX-2 (Absin, abs131986), rabbit anti-IL-1β (Cell Signaling Technology, 12703), rabbit anti-Flag (Proteintech, 20543-1-AP), mouse anti-GAPDH (Proteintech, 60004-1-Ig), rabbit anti-EV71 3D (Abclonal, A22651), and rabbit anti-CA16 VP1 (prepared in our laboratory). Goat anti-mouse IgG-HRP and goat anti-rabbit IgG-HRP (Boster, BA1051 and BA1055) were used as secondary antibodies diluted 1:3,000.

### Compounds and *in vitro* antiviral assay

We used 10 distinct NSAIDs as core scaffolds, connecting them via an amide linker to conjugate either a selenocyanate or a novel trifluoromethylseleno organoselenium moiety. This approach yielded a library of 20 novel NSAID-Se derivatives (19). All synthesized compounds were dissolved in dimethyl sulfoxide (DMSO) to a stock concentration of 20 mM for storage. The representative compound YSN-1-167 (molecular weight: 396.1; purity >90% as determined by HPLC) was additionally prepared at a stock concentration of 100 mM to accommodate the higher concentration requirements in cytotoxicity assays. For experimental use, compounds were diluted to working concentrations in cell culture medium, with the final DMSO concentration maintained below 0.1% (vol/vol) in all assays.

To evaluate the anti-EV71 activity of the above compounds, RD or Vero cells were seeded in 12-well plates at a density of 2.5 × 10⁵ cells per well and cultured overnight. After a 2-h viral infection with EV71-GFP (MOI = 1) or EV71-LYG03 (MOI = 0.5), the cells were gently washed with cold PBS and then treated with test compounds (at various concentrations), the vehicle control, or the positive control ribavirin (80 μM) for an additional 22 h. GFP expression was visualized under fluorescence microscopy, and the percentage of GFP-positive cells was determined by flow cytometry (Symphony A1, BD Biosciences). Furthermore, similarly treated cells were either lysed with RIPA buffer (Beyotime, P0013C) for subsequent viral protein analysis or subjected to TRIzol reagent (Invitrogen, 15596026) for viral RNA extraction and subsequent quantification.

### Western blot analysis

To confirm the effects of the compounds on viral infection and inflammatory response, we analyzed viral proteins and inflammatory response-related proteins by Western blot. Briefly, cells were lysed with RIPA buffer (Beyotime, P1045) containing a cocktail of protease inhibitors, and the protein concentration was determined using a BCA protein assay kit (Boster, AR1189). Subsequently, 20–30 μg of protein was separated via 10% SDS-PAGE and transferred to PVDF membranes. After blocking with 5% skim milk for 2 h, the membranes were incubated with primary antibodies overnight at 4°C. The membranes were then incubated with HRP-conjugated secondary antibodies for 1 h at room temperature. Protein bands were visualized using a ChemiDoc XRS Gel Imaging System (Bio-Rad Laboratories) with ECL enhanced chemiluminescence reagents (Beyotime, P0018M).

### Cytotoxicity assay

RD or Vero cells were seeded into 96-well plates at a density of 1 × 10⁴ cells per well and cultured overnight and treated with various concentrations of YSN-1-167 for 24 h. Then, 10 μL of CCK-8 reagent (Cell Counting Kit-8, C0038, Beyotime) was added to each well and incubated at 37°C for 1 h; the absorbance at 450 nm was measured using a multimode microplate reader (MultiskanTM FC, Thermo Fisher). Cell viability was calculated as the percentage of absorbance of YSN-1-167-treated cells relative to the vehicle control. The 50% cytotoxic concentration (CC₅₀) was calculated using GraphPad Prism 8 software.

### Real-time quantitative PCR

Total RNA was isolated using TRIzol reagent, then 2 μg of RNA was reverse transcribed to cDNA using the HiFiScript gDNA Removal cDNA Synthesis Kit (CWBIO, CW2582M), and the synthesized cDNA was used as the template to perform RT-qPCR using TB Green Premix Ex Taq II FAST qPCR Mix (Takara, RR420Q) on a CFX96 Real-Time PCR Detection System (Bio-Rad Laboratories). The GAPDH gene was used as the internal control. The primer sequences used in this study are listed in Table 1.

**TABLE 1:** The primer sequences used for RT-qPCR analysis in this study

Primer	Sequence
EV71-VP1-F	GCAGCCCAA AAGAACTTCAC
EV71-VP1-R	ATTTCAGCAGCTTGGAGTGC
Human GAPDH-F	GCACCGTCACGGCTGAGAAC
Human GAPDH-R	TGGTGAAGACGCCAG TGGA
Mouse GAPDH-F	TTCACCACCATGGAGAAGGC
Mouse GAPDH-R	GGCATCGACTGTGGTCATGA
Human IL-1β-F	CACGATGCACCTGTACGATCA
Human IL-1β-R	GTTGCTCCATATCCTGTCCCT
Human COX2-F	CTTCCTCCTGTGGCTGATGACTG
Human COX2-R	GGTCCTCGCTTCTGA TCTGTCTTG

### Viral attachment, entry, and replication assay

For the attachment assay, RD cells were pre-chilled at 4°C for 2 h and then incubated with EV71-GFP (MOI = 5) and 50 μM YSN-1-167 at 4°C for 1 h. The cells were then washed three times with ice-cold PBS to remove unbound viruses, followed by RNA extraction and RT-qPCR to detect viral RNA.

For the entry assay, the cells were incubated with EV71-GFP (MOI = 5) at 4°C for 1 h to allow viral attachment. After washing three times with ice-cold PBS to remove unbound viruses, the cells were treated with 50 μM YSN-1-167 and then shifted to 37°C for 1 h to permit viral entry. Finally, the cells were washed three times with PBS and processed for viral RNA detection by RT-qPCR.

For the viral replication assay, cells were infected with EV71-GFP (MOI = 1) at 37°C for 2 h. The viral inoculum was then discarded, and the cells were washed three times with PBS before being cultured in media supplemented with 50 μM YSN-1-167. At 22 h post-infection (hpi), the cells were collected and subjected to viral RNA detection by RT-qPCR and viral protein detection by Western blot.

### Cellular activity assay of EV71 2A protease

The 2A protease disrupts cap-dependent protein synthesis by cleaving elongation factors eIF4GI/eIF4GII, thereby facilitating viral protein production (20). To evaluate whether YSN-1-167 targets 2A protease activity, 293T cells (2 × 10^5^ cells per well in a 12-well plate) were co-transfected with the EV71 2A expression plasmid pFLAG-2A or its catalytically inactive mutant plasmid pFLAG-2A-C110S, along with the reporter plasmid pGLuc using Lipofectamine 2000 Transfection Reagent (Thermo Fisher Scientific, 11,668,030). At 5 h post-transfection (hpt), the Opti-MEM medium was replaced with DMEM containing YSN-1-167 or DMEM containing DMSO (vehicle control). After 48 h, the culture supernatants were collected, and the GLuc activities were measured using a Gaussia Luciferase Reporter Gene Assay Kit (Beyotime, RG021S) according to the manufacturer’s instructions.

### The activity assay of EV71 2A and 3C proteases *in vitro*

EV71 3C and 2A proteases were purified as previously described (21, 22) with minor modifications. Briefly, the genes encoding EV71 3C or 2A protease were amplified and cloned into the pET30a expression vector using BamHI and NotI restriction sites. The recombinant plasmids were transformed into *Escherichia coli* BL21 (DE3), and protein expression was induced with 0.5 mM isopropyl β-D-1-thiogalactopyranoside (IPTG). The recombinant proteases were purified by Ni-NTA affinity chromatography, and were stored at −80°C at a concentration of 1 mg/mL.

Protease activity was assessed using a fluorescence-based cleavage assay. The 2A protease substrate Dabcyl-KSRTAITTLGKFGQQSGE-Edans corresponds to its autocatalytic cleavage site (VP1/2A), while the 3C protease substrate Dabcyl-RTATVQGPSLDFE-Edans represents a Q-G cleavage site (23). Proteolytic cleavage separates the fluorophore (Edans) from the quencher (Dabcyl), generating a fluorescence signal for real-time activity monitoring. The fluorescence intensity directly reflects protease activity and was used to evaluate compound inhibition. Activity assays were performed using 1 μM 2A or 3C protease in 20 mM Tris-HCl (pH 7.5), 100 mM NaCl, 2 mM DTT, and different concentrations of YSN-1-167. Fluorescence was monitored (excitation at 340 nm, emission at 500 nm) using a PerkinElmer VICTOR Nivo multi-mode microplate reader, and the raw fluorescence intensity was recorded for analysis.

### Cellular activity assay of EV71 3D polymerase

To evaluate the effect of YSN-1-167 on EV71 3D^pol^ activity *in vivo*, we established a GLuc reporter system using a chimeric plasmid (designated 3-G-5), wherein the GLuc gene is flanked by the EV71 5′-UTR and 3′-UTR to mimic the structure of viral genomic RNA. This reporter construct was co-transfected with a 3Flag-3D plasmid for the overexpression of EV71 3D^pol^. Mechanistically, 3D^pol^ acts as an RdRp that binds to the EV71 3′-UTR to initiate viral RNA replication and drive the downstream expression of the GLuc gene. At 5 hpt, cells were treated with serial dilutions of YSN-1-167 or the positive control ATA. Culture supernatants were collected at 48 hpt, and the activity of secreted GLuc was quantified using the Gaussia Luciferase Reporter Gene Assay Kit. Notably, GLuc expression levels directly correlate with 3D^pol^ activity, thereby enabling the quantitative assessment of compound-mediated inhibition of viral replication.

### IRES-based bicistronic reporter assay

To evaluate whether YSN-1-167 affects EV71 IRES-dependent translation, we constructed a pFLuc-EV71-IRES reporter plasmid. This plasmid harbors the EV71 5′-UTR containing the IRES upstream of the FLuc open reading frame, thereby positioning FLuc expression under the control of the IRES. The activity of FLuc serves as a direct readout of IRES function, enabling compound inhibition assessment via luminescence measurement. For the assay, 293T cells were seeded in 12-well plates (2 × 10^5^ cells per well) and co-transfected with the reporter plasmid and an internal control plasmid expressing RLuc. At 5 hpt, the Opti-MEM medium was replaced with DMEM containing serial dilutions of YSN-1-167 or positive control sorafenib. After 48 h, cells were lysed with lysis buffer from the Dual-Luciferase Reporter Assay Kit (Vazyme, DL101-01), and the activities of FLuc and RLuc were quantified according to the manufacturer’s protocol. FLuc activity values were normalized to the corresponding RLuc activity for each sample.

### The drug affinity responsive target stability assay

The drug affinity responsive target stability assay was performed following a published protocol (24). Briefly, 293T cells transfected with the 3D^pol^ expression plasmid were lysed with RIPA buffer (Beyotime, P0013D) supplemented with a complete protease inhibitor cocktail. The lysate supernatant was collected and incubated with 50 μM or 150 μM YSN-1-167, vehicle control, or positive control ATA overnight at 4°C, then pronase (25 ng enzyme per μg protein) was added and incubated for 4 min at room temperature. The reactions were terminated by adding SDS loading buffer and heating at 100°C for 10 min, and then were used to detect the stability of 3D with Western blot to speculate the binding ability of YSN-1-167 to 3D.

### Cellular thermal shift assay

The cellular thermal shift assay (CETSA) was performed based on previously described protocols (25) with modifications. 293T cells were seeded in 6 cm culture dishes and cultured overnight, then were transfected with EV71-3D plasmid, and were harvested at 24 hpt using RIPA lysis buffer supplemented with 1% protease inhibitor cocktail. The lysate supernatant was then incubated with 150 μM YSN-1-167 or vehicle control overnight at 4°C. Afterward, the mixture was aliquoted into seven portions in PCR tubes and heated at designated temperatures (37°C, 41°C, 45°C, 49°C, 53°C, 57°C, and 61°C) for 10 min. The samples were subjected to three freeze–thaw cycles and then were separated by centrifugation at 12,000 rpm for 15 min at 4°C, and the supernatants were subjected to Western blot.

### Molecular docking of 3D^pol^ and YSN-1-167

The structure of 3D^pol^ was obtained from the Protein Data Bank (PDB). The structure of the ligand YSN-1-167 was drawn using ChemDraw, followed by energy minimization and protonation, which were performed in Discovery Studio. The optimized structure of YSN-1-167 was locally saved. Docking analysis was performed using AutoDock Tools 4.2, and the docking results were visualized using the PyMOL Molecular Graphics System. The binding interaction of YSN-1-167 with the target protein was evaluated based on the consensus score results.

### Acute toxicity evaluation of YSN-1-167 in neonatal ICR mice

Pregnant ICR mice were obtained from Vital River Laboratories and maintained under specific pathogen-free (SPF) conditions. The 4-day-old ICR mice were randomly assigned to two groups (*n* = 10 per group): a PBS control group and a YSN-1-167-treated group. The *in vivo* dose of YSN-1-167 (14 mg/kg) was determined by converting its maximum safe concentration with effective anti-EV71 function (50 μM) from *in vitro* studies, using a body fluid volume-equivalent approach (26). Mice of the treated group received intraperitoneal (i.p.) injections of YSN-1-167 at 14 mg/kg once daily for seven consecutive days. The injection volume was 20 μL per mouse. Control mice received an equal volume of PBS alone. Body weight was recorded daily. At 24 h after the final injection, all mice were euthanized. Blood was collected for serum separation. Serum levels of alanine aminotransferase (ALT), aspartate aminotransferase (AST), blood urea nitrogen (BUN), and creatinine (CRE) were measured to assess liver and kidney function. The kidney, liver, and spleen were excised and weighed to calculate the organ-to-body weight ratio (organ index). Tissues were then fixed in 4% paraformaldehyde for histopathological analysis with hematoxylin and eosin (H&E) staining. All experimental procedures involving mice were approved by Jianghan University (Approval number: JHDXKJLL2025-174).

### Anti-EV71 activity of YSN-1-167 in neonatal ICR mice

The 3-day-old neonatal mice were randomly divided into three groups (*n* = 6 per group) and received intraperitoneal injections as follows: the control group received PBS; the EV71 infection group received 1 × 10⁶ PFU of EV71-LYG03, followed by PBS injection; and the EV71 infection +YSN-1-167-treatment group received 1 × 10⁶ PFU of EV71-LYG03, followed by 14 mg/kg YSN-1-167 injection. Beginning at 24 hpi, YSN-1-167 was administered daily intraperitoneally for four consecutive days. Mice were euthanized on day 5 post-infection (dpi), and hindlimb muscle tissues were collected for viral load analysis using RT-qPCR and histopathological analysis with H&E staining. All offspring infection experiments were conducted in the Animal Biosafety Level 2 (ABSL-2) facility at the Wuhan Institute of Virology, Chinese Academy of Sciences. All experimental procedures involving mice were approved by the same ethics approval as the above acute toxicity evaluation experiment.

### Statistical analysis

Data are presented as mean ± standard error of the mean (SEM). Statistical significance was analyzed using one-way analysis of variance (ANOVA) with Tukey’s multiple comparison test and the unpaired *t*-test. Differences were considered significant at *P* < 0.05. All analyses were performed using GraphPad Prism 8.0 (GraphPad Software, Inc.).

## RESULTS

### Compound library screening identifies YSN-1-167 as an inhibitor of EV71 infection

**Fig 1:**
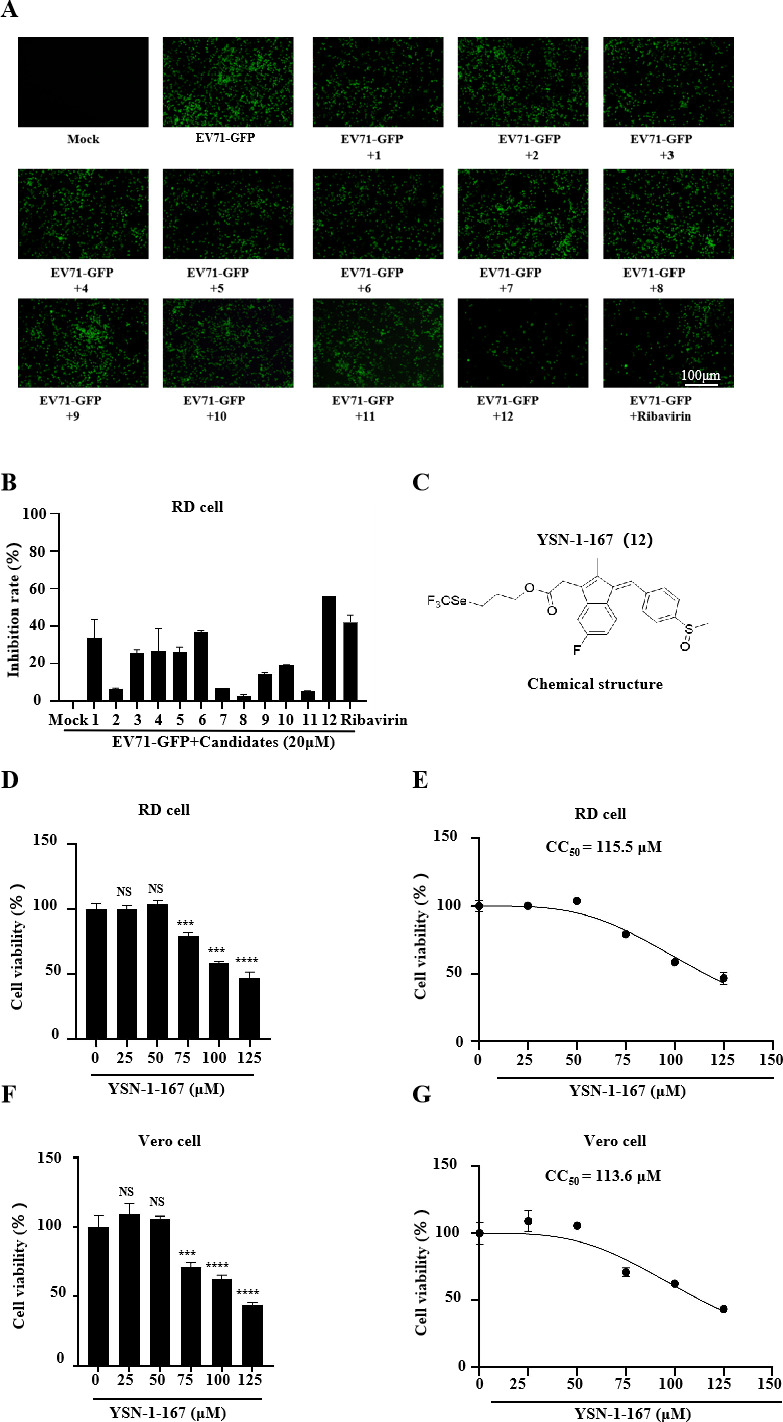
Screening of NSAID-Se derivatives for anti-EV71 activity using a GFP reporter virus. (**A**) Representative fluorescence microscopy images of RD cells infected with EV71-GFP (MOI = 1) and treated with 20 μM NSAID-Se derivatives or 80 μM ribavirin (positive control) at 2 hpi, following 22 h of treatment; scale bar = 100 µm. (**B**) Quantitative analysis of the percentages of GFP-positive cells measured via flow cytometry. Mock: uninfected control. The inhibition rates were calculated using the following formula: ([Percentage of GFP-positive cells in mock group − Percentage in treated group]/Percentage in mock group) × 100%. (**C**) Chemical structure of compound 12. The cytotoxicity analysis of YSN-1-167 in RD (**D, E**) and Vero (**F, G**) cells by CCK-8 assay at 24 h post-treatment. All data are presented as mean ± SEM of triplicate measurements. NS, no significant; ***P < 0.001; ****P < 0.0001.

To identify the compounds with dual functions of anti-inflammatory and anti-EV71 properties, we synthesized a library of 20 NSAID-Se derivatives and conducted a primary screening with an EV71-GFP reporter virus infection in RD cells. Microscopic examination revealed that eight compounds exhibited significant cytotoxicity at the tested concentration (20 μM). The remaining 12 compounds were advanced for further analysis, and the ratio of GFP-positive cells upon treatment with each was quantified by flow cytometry. The results showed that 6 out of 12 compounds exhibited inhibitory activity against EV71, and compound 12 showed the strongest inhibitory effect with an inhibition rate of 55%, which was superior to that of the positive control ribavirin (Fig. 1A and B). The chemical structure of compound 12, designated YSN-1-167, was derived from sulindac (Fig. 1C). We further employed the CCK-8 assay to evaluate the safety concentration of YSN-1-167 in RD and Vero cells. The results showed that YSN-1-167 is safe up to 50 μM in two cell lines, and the CC_50_ of YSN-1-167 was 115.5 μM in RD cells (Fig. 1D and E) and 113.6 μM in Vero cells (Fig. 1F and G). Collectively, the anti-EV71 function and favorable safety of YSN-1-167 in target cells highlight its potential as a promising candidate drug for anti-EV71 infection.

### YSN-1-167 inhibits EV71 infection in a dose-dependent manner

**Fig 2:**
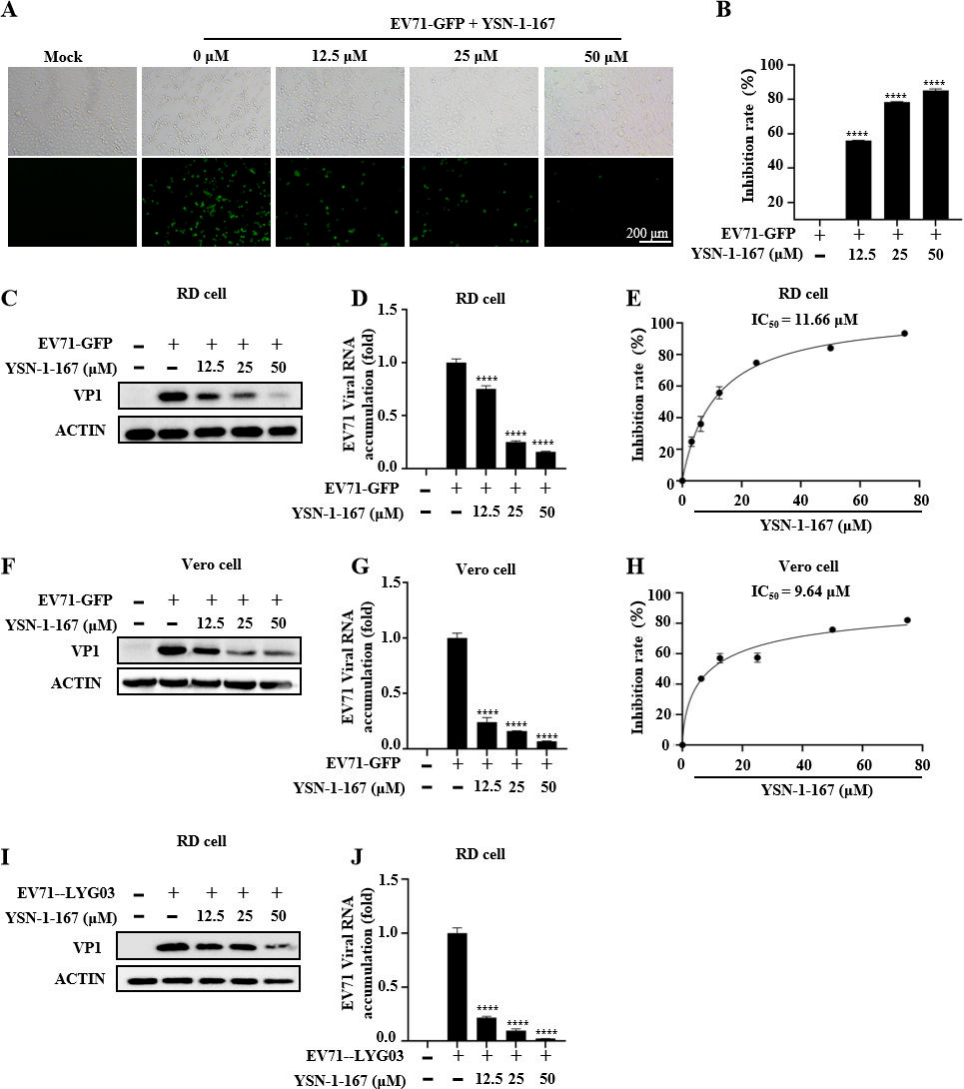
Anti-EV71 activity of YSN-1-167 in RD and Vero cells. (**A**) Representative fluorescence (upper panels) and bright-field (lower panels) images of RD cells, which were infected with EV71-GFP (MOI = 1) and treated with YSN-1-167 (0–50 μM) at 2 hpi, followed by 22 h of incubation; scale bar = 200 µm. And the quantifications of GFP-positive cell ratios were measured by flow cytometry (**B**), and VP1 protein levels were detected by Western blot (**C**), viral RNA was quantified by RT-qPCR (**D**), and the IC₅₀ was calculated from the viral RNA data using GraphPad Prism 8.0 (**E**). Similar experiments were performed in Vero cells, the levels of VP1 protein (**F**) and viral RNA (**G**) were detected, and the IC_50_ of YSN-1-167 in Vero cells was calculated from viral RNA data (**H**). Further confirmation experiments were performed with wild-type EV71-LYG03 in RD cells, and the levels of VP1 protein and viral RNA were detected (**I and J**). Viral RNA levels are normalized to the vehicle control group (set as 1). All data are presented as mean ± SEM of triplicate measurements. NS, no significant, ****P < 0.0001.

To further evaluate the antiviral activity of YSN-1-167, RD cells were infected with EV71-GFP at 2 hpi, and the cells were treated with the compound at concentrations ranging from 0 to 50 μM for 22 h. The observation of CPE and GFP fluorescence showed that YSN-1-167 inhibited viral infection in a concentration-dependent manner (Fig. 2A and B), as evidenced by the decreased viral VP1 protein levels (Fig. 2C). The viral RNA detection also demonstrated that YSN-1-167 significantly inhibited EV71-GFP infection, from which half maximal inhibitory concentration (IC_50_) was calculated to be 11.66 μM (Fig. 2E). A similar dose-dependent suppression of VP1 protein (Fig. 2F) and viral RNA (Fig. 2G) was confirmed in Vero cells, with an IC_50_ of 9.64 μM calculated from the viral RNA data (Fig. 2H). The selectivity index (SI) of YSN-1-167 against EV71 in RD and Vero cells is shown in Table S1. Furthermore, the antiviral efficacy of YSN-1-167 was validated against a wild-type EV71-LYG03 strain in RD cells; the results confirmed the concentration-dependent reductions in the levels of viral RNA and VP1 protein (Fig. 2I and J). These findings conclusively demonstrated that YSN-1-167 is a potent inhibitor of EV71 infection.

### YSN-1-167 exerts an antiviral effect by targeting the replication stage of the EV71 life cycle

**Fig 3:**
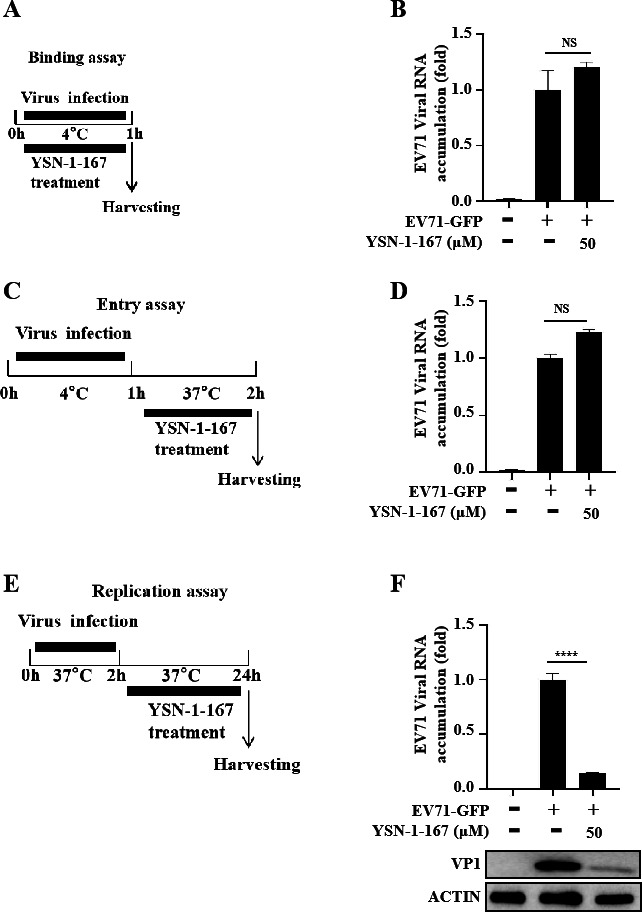
The effect of YSN-1-167 in the life cycle of EV71. The schematic of the attachment assay (**A**). Pre-chilled RD cells were incubated with a mixture of EV71-GFP (MOI = 5) and 50 μM YSN-1-167 for 1 h at 4°C. After washing with ice-cold PBS, viral RNA from bound viruses was quantified by RT-qPCR (**B**). The schematic of the entry assay (**C**). Following the attachment of EV71-GFP (MOI = 5) to RD cells at 4°C for 1 h, cells were washed with PBS and treated with 50 μM YSN-1-167 at 37°C for 1 h, and intracellular viral RNA was quantified by RT-qPCR (**D**). The schematic of the replication assay (**E**). After infection with EV71-GFP (MOI = 1) for 2 h at 37°C, RD cells were treated with 50 μM YSN-1-167 for another 22 h. The levels of viral RNA (RT-qPCR) and viral protein VP1 (Western blot) were analyzed (**F**). Viral RNA levels are normalized to the vehicle control group (set as 1). All data are presented as the mean ± SEM of triplicate measurements. NS, no significant; ****P < 0.0001.

To determine which stage of the EV71 life cycle is targeted by YSN-1-167, we performed a time-of-addition assay to investigate the effect of YSN-1-167 on the attachment, entry, and replication stages of the virus. For the attachment assay, RD cells were incubated with the mixture of EV71-GFP and YSN-1-167 for 1 h at 4°C to allow virus binding but not internalization (Fig. 3A). Subsequent RT-qPCR analysis for intracellular viral RNA revealed that YSN-1-167 did not affect the attachment of EV71 to host cells (Fig. 3B). RD cells with pre-attached virus at 4°C for 1 h were treated with YSN-1-167 at 37°C for 1 h to permit synchronous viral entry. Intracellular viral RNA quantification revealed that YSN-1-167 also did not affect the entry of EV71 into host cells (Fig. 3C and D). In contrast, for the replication assay, RD cells were infected with EV71-GFP for 2 h at 37°C, then washed to remove unbound virus, and treated with YSN-1-167 for 22 h (Fig. 3E). In YSN-1-167-treated cells, the levels of viral RNA and VP1 protein significantly decreased compared with vehicle-treated cells (Fig. 3F). These findings indicated that YSN-1-167 specifically interferes with the replication of EV71 but not the attachment and entry stages.

Given the pivotal roles of viral proteases 2A/3C, the viral polymerase 3D, and IRES-mediated polyprotein translation during the viral replication stage, we further systematically examined the effect of YSN-1-167 on these three key targets of the replication stages: (i) the proteolytic activity of the viral 2A and 3C proteases, (ii) the RNA polymerization activity of the viral 3D^pol^, and (iii) IRES-mediated polyprotein translation.

### YSN-1-167 inhibits EV71 through direct suppression of 3D^pol^ activity

**Fig 4:**
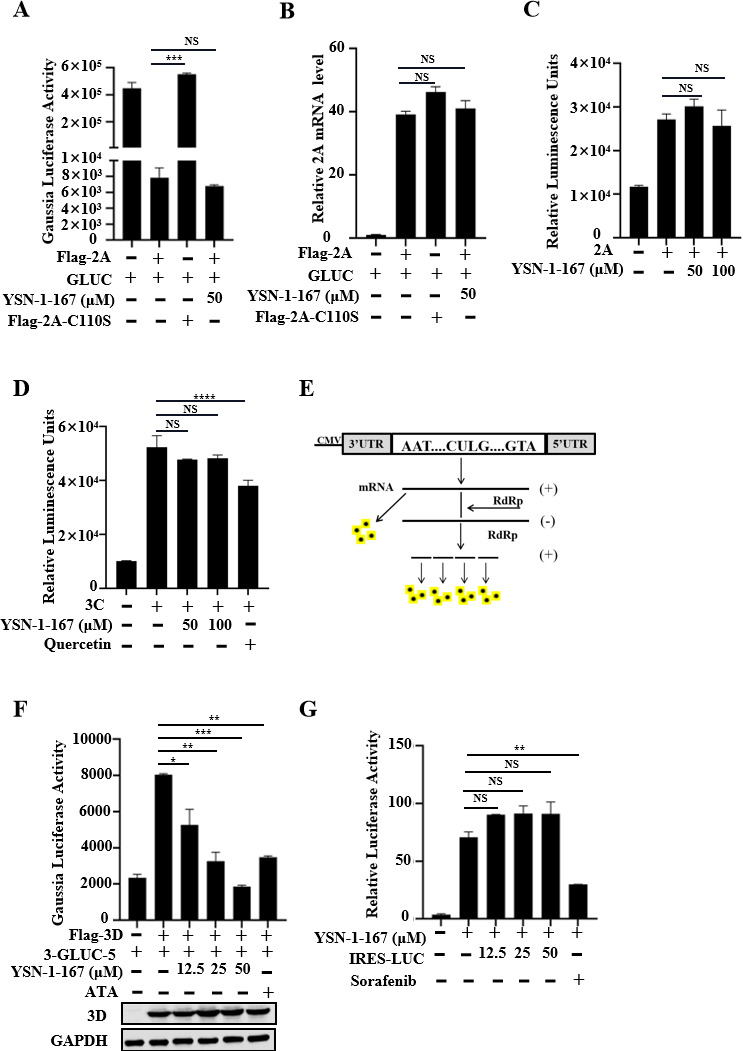
The target investigation of YSN-1-167 in EV71 replication. 293T cells were co-transfected with pGLuc reporter and pFLAG-2A or pFLAG-2A-C110S plasmids, treated with 50 μM YSN-1-167 or vehicle at 5 hpt, and GLuc activity was quantified at 48 h to evaluate the activity of 2A (**A**), and the expression levels of pFLAG-2A and its mutant were detected by RT-qPCR (**B**). The purified 2A and 3C proteins were incubated with their respective substrates for 6 h in the presence of YSN-1-167 (50 or 100 μM), vehicle control, and quercetin was used as a positive control for 3C (100 μM). Proteolytic cleavage activity was quantified by fluorescence intensity (**C and D**). (**E**) The schematic diagram of the 3D^pol^-dependent Gaussia luciferase reporter system. (**F**) 293T cells were co-transfected with EV71 3D^pol^ and the reporter plasmid 3-G-5 plasmids. At 5 hpt, the cells were treated with a concentration gradient of YSN-1-167 (0–50 μM) or 200 μM ATA (positive control). Culture supernatants were collected after 48 h for GLuc activity quantification to evaluate the activity of 3D. (**G**) 293T cells were transfected with the pFLuc-EV71-IRES reporter plasmid and treated with YSN-1-167 (0–50 μM) or with a positive control, sorafenib (3 μM), for 48 h. FLuc and Renilla luciferase (RLuc) activities in the cell lysates were quantified. All data are mean ± SEM of triplicates. NS, no significant; **P < 0.01; ***P < 0.001; ****P < 0.0001.

To assess the effect of YSN-1-167 on EV71 2A protease activity, we first performed a cellular reporter assay in 293T cells. The Gluc activity detection showed that the expression of pFLAG-2A, but not the inactive mutant pFLAG-2A-C110S, significantly reduced the GLuc signal, confirming the validity of the assay system that 2A has the protease activity. Importantly, YSN-1-167 treatment failed to rescue the GLuc signal in pFLAG-2A-expressing cells, indicating no inhibitory effect on the proteolytic activity of EV71 2A protein (Fig. 4A and B). We further validated this finding *in vitro* using a fluorogenic cleavage assay, and the results confirmed that YSN-1-167 did not alter the proteolytic activity of purified 2A protein (Fig. 4C). We also performed a similar *in vitro* experiment on EV71 3C protease; the results showed that YSN-1-167 also did not affect the proteolytic activity of purified 3C protein, whereas the positive control quercetin (100 μM) markedly suppressed its activity (Fig. 4D).

Having excluded 2A and 3C proteases as potential antiviral targets of YSN-1-167, we next examined whether the compound targets EV71 RNA-dependent RNA polymerase 3D^pol^, a core enzyme mediating viral genome replication. A custom-constructed 3D^pol^-dependent GLuc reporter plasmid 3-G-5 (Fig. 4E) was co-transfected with a 3D^pol^ expression plasmid into 293T cells; the expressed 3D^pol^ acts as an RdRp to replicate the reporter RNA and thereby enhance GLuc gene expression. The detection results showed that YSN-1-167 (0–50 μM) significantly suppressed GLuc activity in a dose-dependent manner, and its inhibitory effect was more potent at 50 μM than that of the positive control, ATA (Fig. 4F). Since the 3-G-5 reporter system relies not only on 3D^pol^-mediated RNA replication but also on the EV71 IRES element in its 5′-UTR for cap-independent translation, we further detected the potential impact of YSN-1-167 on IRES-mediated translation using an independent EV71 IRES-driven FLuc reporter assay. Notably, while the positive control, sorafenib, strongly suppressed IRES-mediated FLuc activity, YSN-1-167 showed no inhibitory effect even at the maximum concentration of 50 μM (Fig. 4G). These results suggest that YSN-1-167 might specifically target 3D^pol^ activity.

In addition, to demonstrate the specific inhibition function of YSN-1-167 on EV71, we examined the inhibitory effect of YSN-1-167 on other RNA viruses, including the negative-sense RNA virus, influenza PR8, and the positive-sense RNA virus, dengue virus. The results showed that YSN-1-167 at 50 μM did not inhibit the replication of these viruses (Fig. S1A and B), which further confirmed the specificity suppression of YSN-1-167 on EV71 3D^pol^ activity. Collectively, these results definitively demonstrate that YSN-1-167 exerts its anti-EV71 activity by specifically suppressing the enzymatic activity of 3D^pol^, not by inhibiting 2A/3C protease activity or interfering with IRES-mediated translation.

Given the high sequence conservation among enteroviruses, we performed a comparative analysis of the EV71 3D^pol^ with its homologs from representative strains of Enterovirus A, B, C, and D according to the established molecular genotyping system. The results showed that the 3D^pol^ of CA16 (also a strain of Enterovirus A) exhibited extremely high sequence identity with that of EV71, whereas its identity with representative strains from Enterovirus B, C, and D was only approximately 60% (Fig. S2A). Therefore, we further investigated the antiviral activity of YSN-1-167 to CA16. The results showed that YSN-1-167 attenuated CA16-induced CPE (Fig. S2B) and decreased the levels of VP1 protein and viral RNA of CA16 in a dose-dependent manner (Fig. S2C and D). An IC₅₀ of 0.55 μM for CA16 inhibition was calculated from the viral RNA data (Fig. S2E). These results demonstrated that YSN-1-167 effectively inhibits both EV71 and CA16 infection, likely by targeting 3D^pol^ to interfere with viral RNA replication.

### YSN-1-167 might interact with EV71 3D^pol^

**Fig 5:**
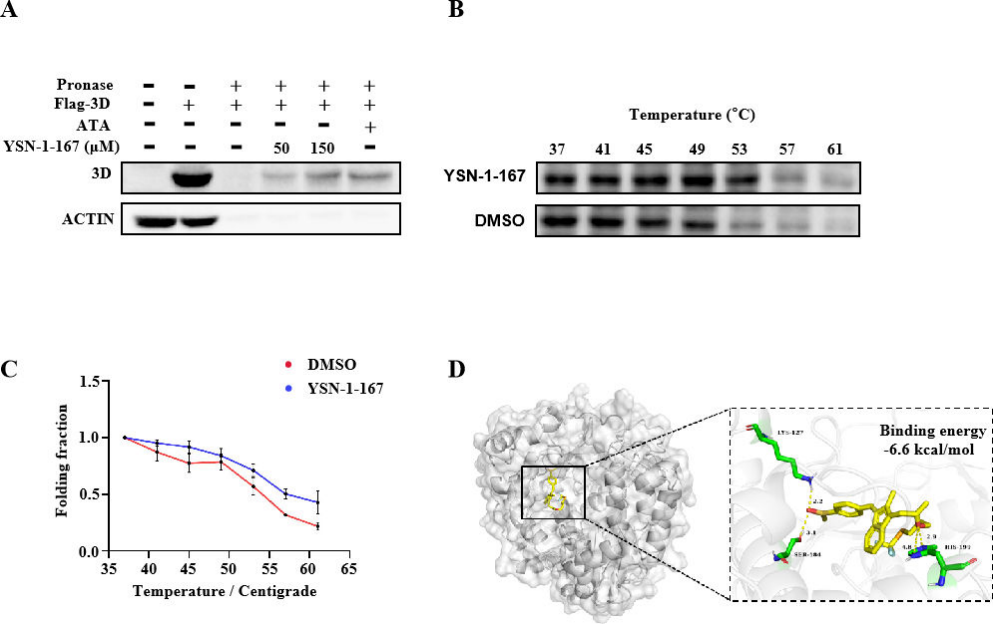
The effect of YSN-1-167 on EV71 3D^pol^. (**A**) The lysates from 3D^pol^-transfected 293T cells were incubated with 50 or 150 μM YSN-1-167, vehicle, or 200 μM ATA (positive control), followed by limited proteolysis treatment with 25 ng pronase per 1 μg protein for 4 min. The stability of 3D^pol^ was assessed by Western blot. (**B, C**) The lysates from 3D^pol^-transfected 293T cells containing 150 μM YSN-1-167 or vehicle (incubated overnight at 4°C) were heated at the indicated temperatures for 10 min, then the protein level of 3D^pol^ was analyzed by Western blot, and the corresponding melting curve was plotted based on protein band intensities. (**D**) The ligand structure was prepared using ChemDraw, followed by energy minimization and protonation in Discovery Studio, and molecular docking was performed using AutoDock Tools 4.2; the docking results were visualized using PyMOL.

To investigate whether YSN-1-167 interacts with the EV71 3D^pol^, we performed a drug affinity responsive target stability (DARTS) assay. Cell lysates containing expressed 3D^pol^ were incubated with YSN-1-167 or vehicle, then treated with pronase, and the protein levels of 3D^pol^ were detected by Western blot. The results showed that YSN-1-167 can protect 3D^pol^ from degradation by pronase in a concentration-dependent manner, with a protective effect comparable to the positive control, ATA (Fig. 5A). To further validate this interaction, we performed CETSA using 293 T cell lysates containing 3D^pol^ protein. The lysates were incubated with YSN-1-167 or vehicle, followed by heating at different temperatures. As shown in Fig. 5B and C, YSN-1-167 treatment increased the thermal stability of 3D^pol^ compared to the vehicle control, which suggests that YSN-1-167 might bind to 3Dpol. These results from DARTS and CETSA assays provide preliminary evidence for an interaction between YSN-1-167 and 3D^pol^. Molecular docking simulations were then performed to characterize the potential binding mode. The results suggested that YSN-1-167 potentially binds to the catalytic pocket of 3D^pol^ via its selenium atom with a calculated binding energy of −6.6 kcal/mol (Fig. 5D). Collectively, these findings suggest that YSN-1-167 exerts anti-EV71 activity by inhibiting its polymerase activity, potentially through interacting with the 3D protein.

### YSN-1-167 significantly suppresses the inflammatory response induced by LPS treatment and EV71 infection

**Fig 6:**
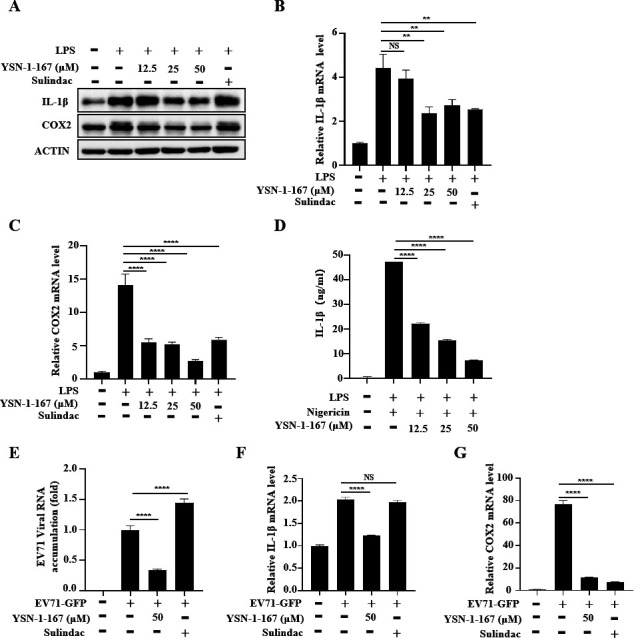
Anti-inflammatory activity of YSN-1-167. PMA-differentiated THP-1 cells were pretreated with the indicated concentrations of YSN-1-167 or sulindac for 12 h, followed by stimulation with LPS (1 μg/mL), the protein levels of IL-1β and COX-2 were detected by Western blot (**A**), and the mRNA levels of IL-1β and COX-2 were quantified by RT-qPCR (**B and C**). After LPS priming and nigericin (10 μM) stimulation for inflammasome activation, the secreted IL-1β level was measured by ELISA (**D**). PMA-differentiated THP-1 cells were infected with EV71-GFP at MOI = 10 for 2 h, then were treated with compounds for another 22 h. The cellular RNA was extracted, and the intracellular viral RNA (**E**) and the mRNA levels of IL-1β and COX-2 (**F, G**) were detected by RT-qPCR. Viral RNA levels were normalized to the vehicle group (set as 1). All data are presented as the mean ± SEM of triplicate measurements. NS, no significant; ** *P* < 0.01; ****P < 0.0001.

Based on its molecular design as an NSAID derivative, we hypothesized that YSN-1-167 might possess anti-inflammatory activity. We first investigated this function in the PMA-differentiated THP-1 macrophage cell line; the cells were pretreated with YSN-1-167 for 12 h prior to stimulation with LPS (1 μg/mL); an anti-inflammatory compound, sulindac, was used as a positive control. Analysis of key inflammatory mediators showed that YSN-1-167 significantly reduced both the protein (Fig. 6A) and mRNA (Fig. 6B and C) levels of IL-1β and COX-2. Furthermore, we measured the secreted IL-1β by ELISA following LPS priming and nigericin-triggered inflammasome activation, and the results showed that YSN-1-167 markedly decreased the level of secreted IL-1β (Fig. 6D).

As it is known, EV71 infection induces an inflammatory response. Therefore, we investigated the anti-inflammatory effect of YSN-1-167 in EV71-infected cells. An EV71-GFP infection model (MOI = 10) was established in PMA-differentiated THP-1 cells to explore potential antiviral and anti-inflammatory synergy; the results showed that YSN-1-167, but not sulindac, significantly reduced intracellular viral RNA levels (Fig. 6E) and decreased the mRNA levels of IL-1β and COX-2 induced by EV71 infection, and sulindac significantly inhibited COX-2 expression but did not affect IL-1β levels (Fig. 6F and G). Collectively, these results demonstrated that YSN-1-167 retains anti-inflammatory functions akin to NSAIDs and can inhibit the inflammatory response induced by EV71 infection.

### YSN-1-167 shows favorable safety in an acute toxicity study in mice

**Fig 7:**
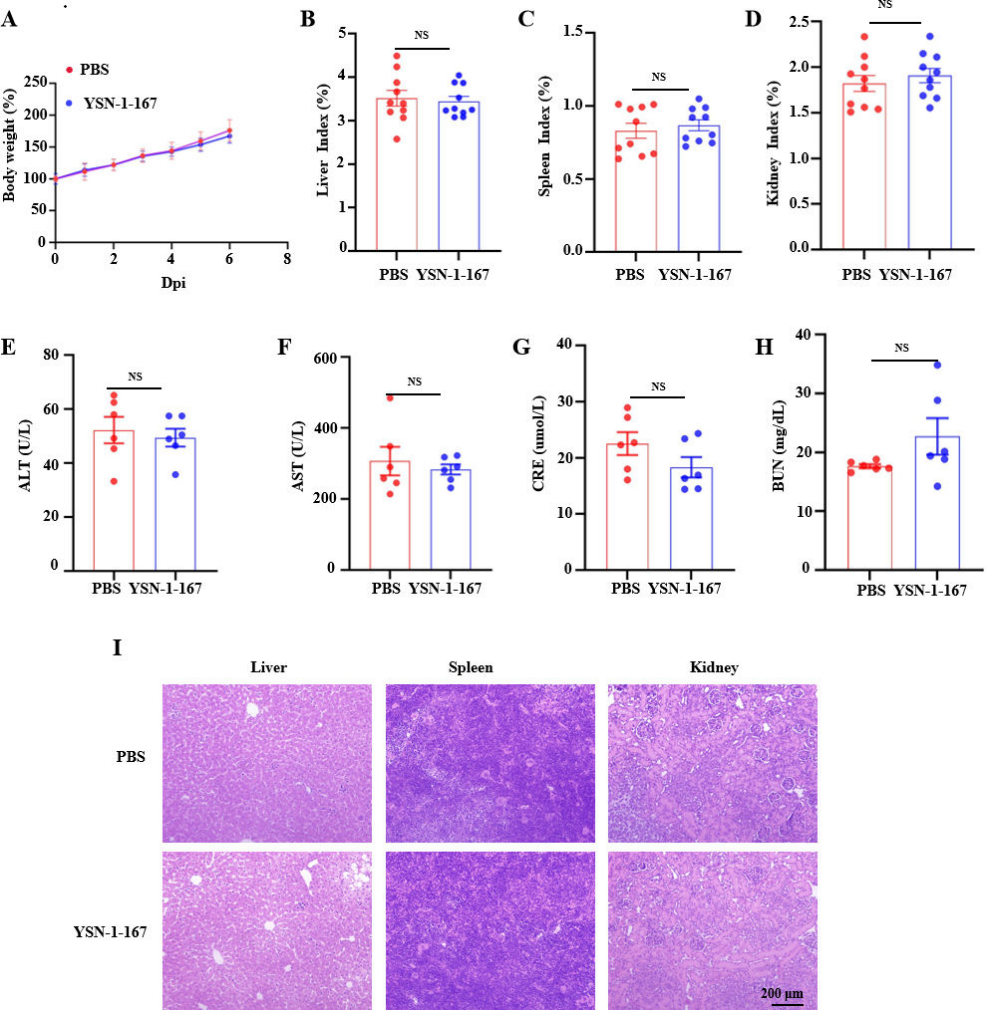
Evaluation of the acute toxicity of YSN-1-167 in neonatal ICR mice. Four-day-old ICR mice were treated with YSN-1-167 (14 mg/kg/day, i.p.) or PBS for seven consecutive days. Body weight changes were monitored daily (**A**). Relative organ-to-body weight ratios of kidney (**B**), liver (**C**), and spleen (**D**) were calculated at 24 h after the final injection. Serum biochemical parameters of ALT (**E**), AST (**F**), CRE (**G**), and BUN (**H**) were measured. Representative histopathological images of liver, kidney, and spleen by H&E staining are shown (**I**); scale bar = 200 µm. *N* = 10. Data are the means ± SEM. NS, no significant.

To assess the potential acute toxicity of YSN-1-167, newborn ICR mice were administered YSN-1-167 or PBS for seven consecutive days, with daily body weight monitoring. The results showed a comparable rate of weight gain between the YSN-1-167-treated and PBS control groups, indicating no adverse effects on growth and development (Fig. 7A). All mice were euthanized for analysis at 24 h after the final injection, and the kidneys, liver, and spleen were collected and weighed. The organ-to-body weight ratios showed no significant differences compared with those of the PBS group (Fig. 7B through D), suggesting an absence of treatment-induced organ swelling or atrophy. Serum biochemical analysis demonstrated that the markers of liver function (ALT and AST) and kidney function (CRE and BUN) remained within normal ranges following YSN-1-167 treatment (Fig. 7E through H), indicating a lack of appreciable hepatotoxicity and nephrotoxicity. Furthermore, histopathological examination via H&E staining revealed no structural abnormalities, inflammatory infiltrates, or tissue damage in the liver, kidney, and spleen of treated mice (Fig. 7I). Collectively, these results indicated that YSN-1-167 treatment at this dosage and duration induces no detectable acute toxicity in neonatal ICR mice, supporting its favorable safety profile for further antiviral investigation *in vivo*.

### YSN-1-167 exerts anti-EV71 activity in mice

**Fig 8:**
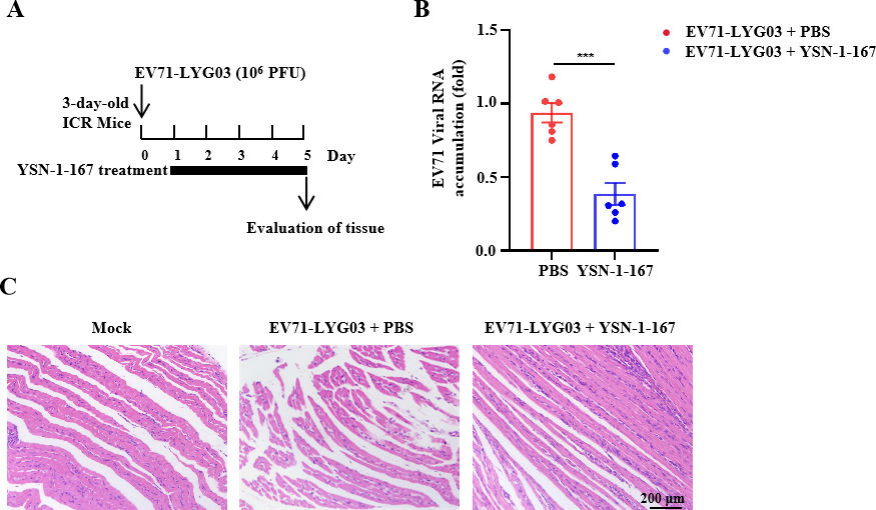
*In vivo* evaluation of antiviral efficacy of YSN-1-167 to EV71 in a neonatal mouse model. (**A**) The schematic diagram of the experimental timeline: newborn mice (3-day-old) were infected with 1 × 10^6^ PFU EV71-LYG03 via i.p. injection. Mice in treatment groups received daily i.p. injections of 14 mg/kg YSN-1-167 (dpi 1–4), and the control group received daily i.p. injections of PBS (Mock: uninfected control; sham: PBS). Muscle tissues were harvested for viral RNA detection by RT-qPCR at 5 dpi (**B**) and for histopathological examination by H&E staining (**C**); scale bar = 200 µm. Viral RNA levels were normalized to the PBS-treated, EV71-infected control group (set as 1). *N* = 6. Data are the mean ± SEM. ***P < 0.001.

To evaluate the therapeutic potential of YSN-1-167 *in vivo*, newborn ICR mice were infected with EV71-LYG03 and treated with YSN-1-167 or PBS (Fig. 8A). All mice were sacrificed at 5 dpi, and viral loads in the muscles were determined by RT-qPCR. The results showed that YSN-1-167 significantly reduced viral RNA accumulation in the muscles (Fig. 8B). Histopathological assessment by H&E staining further revealed that muscle sections from virus-infected mice exhibited severe myonecrosis, inflammatory cell infiltration, and loss of striated muscle architecture. In contrast, YSN-1-167-treated mice displayed markedly preserved tissue integrity, with minimal inflammatory foci and intact muscle fibers, closely resembling the physiological structure observed in the mock group (Fig. 8C). These findings demonstrated that YSN-1-167 effectively inhibits EV71 replication *in vivo* and alleviates virus-induced muscle damage.

## DISCUSSION

In this study, we screened a library of NSAID-derived selenocyanate compounds and identified YSN-1-167 as a potent inhibitor of both EV71 and CA16, exhibiting dose-dependent antiviral activity. Mechanism of action studies revealed that YSN-1-167 specifically suppresses the activity of the viral RNA-dependent RNA polymerase 3D while showing no effect on 2A/3C protease activity or IRES-mediated translation. In line with its NSAID-based design, YSN-1-167 also showed significant anti-inflammatory activity.

Selenium, an essential trace element, is reported to contain multiple physiological functions, and several reports demonstrated that organoselenium has anti-virus functions. Ebselen, an anti-inflammatory organoselenium, can inhibit SARS-CoV-2 replication by binding to the main protease of the virus (11). More recently, ebselen has been shown to exert broad-spectrum antiviral activity against dengue virus, Zika virus, chikungunya virus, EV71, and influenza virus (27). Selenoesters have also been reported to exert potent antiviral activity against herpes simplex virus type 2 (28). Our screening of the organoselenium compound library revealed that several compounds have anti-EV71 activity, but not all derivatives possessed inhibitory effects, suggesting that their antiviral activity is structure-dependent. Replicative enzyme of EV71 screening and molecular docking data indicate that YSN-1-167 might specifically bind to the catalytic pocket of the 3D^pol^ via its selenium atom; whether they are directly binding needs further experiments to confirm.

3D^pol^ of EV71 plays the RdRp function, which is the viral enzyme responsible for viral RNA synthesis, a prominent target for antiviral drug development, as exemplified by the RdRp inhibitor molnupiravir for SARS-CoV-2 and galidesivir for flaviviruses (29) in clinical treatment. Our study demonstrated that YSN-1-167 suppressing EV71 replication might be achieved by interaction with 3D^pol^ to inhibit its enzymatic activity. Since HFMD is induced by multiple viral strains, we compared the full-length 3D^pol^ RNA sequence homologs of other representative strains with EV71 and CA16 of enterovirus A. The results showed that 3D^pol^ of CA16 exhibits high sequence conservation with that of EV71, while the sequence conservation with homologs from Enterovirus B, C, and D is only approximately 60%. We also found that YSN-1-167 also inhibits CA16 replication in a dose-dependent manner, and the IC_50_ is even lower than that of EV71. Recently, it is reported that the prevalence of other Coxsackieviruses such as CA6 and CA10 is increasing; further studies should be performed to evaluate whether YSN-1-167 acts against a broader range of enterovirus A to fully establish its antiviral spectrum and clinical relevance. And toxicological experiments were performed in cells and mice. We found that YSN-1-167 is safe in RD and Vero cells up to 50 μM; according to this concentration, we calculated the safe therapeutic dose in mice. The acute toxicity experiment indicated that a therapeutic dose of YSN-1-167 does not impair liver and kidney function in neonatal ICR mice, underscoring its potential for treating enterovirus infections.

While the inflammatory response is crucial for the body to combat viral infections, excessive systemic inflammation can cause tissue damage and contribute to the pathogenesis of enterovirus infection. An optimal therapeutic strategy involving controlling both viral replication and the inflammatory response using a single agent is expected (30). Studies have reported that EV71 infection upregulates COX-2 expression, and the resulting prostaglandin E2 (PGE2) production, in turn, promotes viral replication, creating a vicious cycle that exacerbates disease. Therefore, targeting COX-2 and its upstream signaling components presents a promising therapeutic approach (31). COX-2 expression is regulated by several transcription factors, including the cyclic-AMP response element-binding protein (CREB), NF-κB, and the CCAAT-enhancer binding protein (C/EBP) (32), and conventional NSAIDs can nonspecifically inhibit COX at standard anti-inflammatory doses (33). As YSN-1-167 was synthesized from sulindac, which can decrease the expression of COX-2 (34), we also detected the anti-inflammation function of YSN-1-167 in the inflammation cell model and EV71-infected macrophages. Our results confirmed that sulindac suppresses the inflammatory response but lacks antiviral activity, whereas YSN-1-167 showed excellent dual functionality, inhibiting both viral replication and COX-2 expression.

Overall, we identified YSN-1-167 as a promising candidate for developing specific therapeutics against HFMD caused by EV71 and CA16 infections, and found that this compound exhibits dual inhibitory functions on both viral replication and inflammatory responses. We further demonstrated that the mechanism underlying its efficacy is that YSN-1-167 inhibits viral replication by suppressing the activity of viral RdRp 3D^pol^ and inhibits the inflammatory response by reducing the expression of both COX-2 and IL-1β. To date, no FDA-approved specific drugs for HFMD are available, and our findings presented here provide valuable clues for further research and for the design of new, effective compounds for HFMD treatment.

## SUPPLEMENTAL MATERIAL

Supplemental material aac.01864-25-s0001.docx Fig. S1 and S2; Table S1.

ASM does not own the copyrights to Supplemental Material that may be linked to, or accessed through, an article. The authors have granted ASM a non-exclusive, world-wide license to publish the Supplemental Material files. Please contact the corresponding author directly for reuse.
