# Supplementary material for: Novel NSAID-Se derivative YSN-1-167 against Enterovirus 71 infection by inhibiting 3Dpol activity
Source: Antimicrob Agents Chemother. 2026 May 29;70(7):e01864-25. doi: 10.1128/aac.01864-25 (PMC13321820; doi:10.1128/aac.01864-25)
Supplement: Supplemental material — Fig. S1 and S2; Table S1. [file aac.01864-25-s0001.docx]

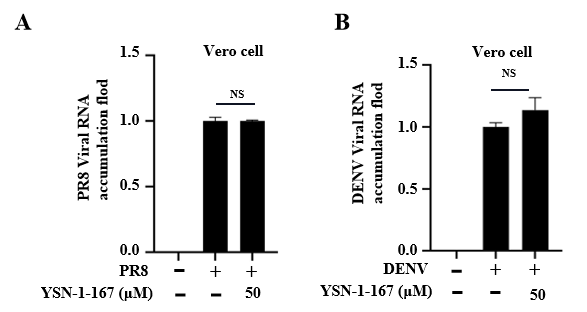


Fig S1. The effect of YSN-1-167 on the replication of influenza virus and dengue virus. Vero cells were infected with influenza virus PR8 (MOI = 0.5) (A) or with dengue virus (MOI = 1) (B) for 2 h, and treated with 50 µM YSN-1-167 for another 46 h. Then the cellular RNA were extracted and the intracellular viral RNA was quantified by RT-qPCR. Viral RNA levels are normalized to the vehicle control group (set as 1). All data are presented as mean ± SEM of triplicate measurements. NS, no significant.


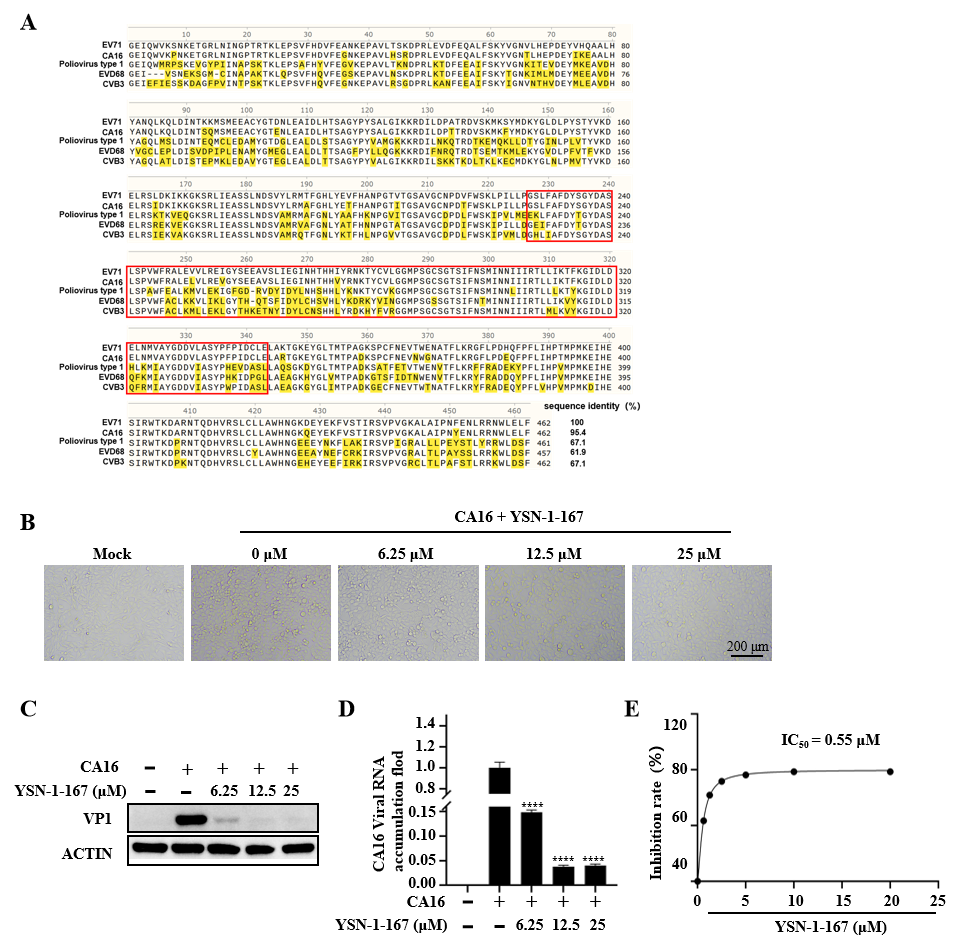


Fig S2. The effect of YSN-1-167 on the replication of CA16. The sequence alignment of full-length 3D^pol^ from EV71 and representative Enterovirus A-D strains, the amino acid residues with variations relative to EV71 are highlighted in yellow, and the enzymatic active sites of 3D^pol^ are boxed in red for clear identification (A). Vero cells were infected with CA16 (MOI = 1) for 2 h, and treated with gradient concentrations of YSN-1-167 (0 to 25 µM) for another 22 h, then the representative images of virus-induced CPE were captured, scale bar = 200 µm (B). Viral VP1 protein expression levels were analyzed by Western blot (C), and intracellular viral RNA was quantified by RT-qPCR (D), the IC_50_ value for YSN-1-167 to CA16 was calculated from the viral RNA data using GraphPad Prism 8.0 (E). Viral RNA normalized to the vehicle control group (set as 1). All data are presented as mean ± SEM of triplicate measurements. NS, no significant; *****P* < 0.0001.

Table S1. Antiviral potency and the selectivity indexes of YSN-1-167 to EV71

|  | CC_50_ (μM) | IC_50_ (μM) | SI |
| --- | --- | --- | --- |
| RD | 115.5 | 11.66 | 9.9 |
| Vero | 113.6 | 9.64 | 11.7 |
